# PPAR-*γ* Thiazolidinedione Agonists and Immunotherapy in the Treatment of Brain Tumors

**DOI:** 10.1155/2008/547470

**Published:** 2008-05-22

**Authors:** Terry Lichtor, Alessandra Spagnolo, Roberta P. Glick, Douglas L. Feinstein

**Affiliations:** ^1^Department of Neurological Surgery, Rush University Medical Center, Chicago, IL 60612, USA; ^2^Department of Anesthesiology, University of Illinois at Chicago, Chicago, IL 60612, USA; ^3^Division of Neurosurgery, Mount Sinai Hospital, New York, NY 10029, USA; ^4^Department of Anatomy and Cell Biology, University of Illinois at Chicago, Chicago, IL 60612, USA

## Abstract

Thiazolidinediones (TZDs) are selective agonists of the peroxisome proliferator-activated receptor (PPAR) gamma, a transcription factor belonging to the superfamily of nuclear hormone receptors. Although activation of PPAR*γ* by TZDs has been best characterized by its ability to regulate expression of genes associated with lipid metabolism, PPAR*γ* agonists have other physiological effects including modulating pro- and anti-inflammatory gene expression and inducing apoptosis in several cell types including glioma cells and cell lines. Immunotherapeutic approaches to reducing brain tumors are focused on means to reduce the immunosuppressive responses of tumors which dampen the ability of cytotoxic T-lymphocytes to kill tumors. Initial studies from our lab show that combination of an immunotherapeutic strategy with TZD treatment provides synergistic benefit in animals with implanted tumors. The potential of this combined approach for treatment of brain tumors is reviewed in this report.

Thiazolidinediones (TZDs) are synthetic compounds originally designed as oral antidiabetic drugs [1, 2], but several reports have indicated other potent biological effects as anti-inflammatory agents [3, 4] and regulators of cell survival [5]. It has been shown that TZDs induce growth arrest and cell death in a broad spectrum of tumor cells [6–10] and hematopoietic cells in vitro and in vivo [11]. At the molecular level, TZDs are specific ligands of the peroxisome proliferator-activated receptor gamma (PPAR-*γ*), a member of the nuclear hormone receptor family. PPAR-*γ* is expressed in many normal tissues, with the highest levels in adipocytes, consistent with its role in lipid metabolism and adipocyte differentiation [2]. Although the ability of TZDs to induce PPAR-*γ* mediated cell differentiation has been clearly demonstrated [12–14], the molecular mechanisms responsible for the growth inhibitory effects of these PPAR-*γ* ligands have not been established [15]. TZDs also bind to a receptor present on the outer mitochondrial membrane termed “mitoNEET” [16] which may mediate some of their metabolic effects and also contribute to induction of apoptosis in tumor cells [17].

Considerable interest has been focused on PPAR-*γ* ligands as potential therapeutic agents in the treatment of gliomas. It has been shown that PPAR-*γ* ligands can induce death in both rodent and human glioma cell lines [18–28]. The antineoplastic effects of TZDs have been related to the ability of these drugs to activate apoptotic pathways [29, 30] or to interfere with the cell cycle through downregulation of cyclin D1 [31] and the upregulation of CDK inhibitors [32, pages (21, 27)]. Interestingly, some studies [24, 27, 33] have directly compared the effects of TZDs on primary astrocytes versus transformed cells, with contrasting results. Two studies [20, 25] showed that ciglitazone, a TZD PPAR-*γ* agonist, was toxic to glioma cells as well as to primary astrocytes, whereas in a third study [27] no toxicity was induced by ciglitazone in normal astrocytes after eight days of incubation. The basis for differential sensitivity of transformed versus nontransformed cells to TZDs is not well understood but may involve differences in metabolic responses [33].

There is some evidence suggesting that PPAR-*γ* also has an immunomodulatory role. In particular, it has been reported that TZDs mediate significant inhibition of proliferative responses of both T cell clones and splenocytes [34]. This inhibition occurs in part because the ligands for PPAR-*γ* mediate inhibition of interleukin-2 (IL-2) secretion by T cell clones while not inhibiting IL-2 induced proliferation of such clones. It has also recently been demonstrated that PPAR-*γ* is a negative regulator of dendritic cell maturation and function [35]. Sustained PPAR-*γ* activation in murine dendritic cell reduced maturation-induced expression of costimulatory molecules and IL-12 and profoundly inhibited their capacity to prime naïve CD4^+^ T cells. Finally, there is some evidence to suggest that TZDs are potent inhibitors of glioma cell migration and brain invasion largely by transcriptional repression of TGF-*β* [36]. This is particularly important because TGF-*β* is an immunosuppressive cytokine that has been shown to have a major role in the malignant phenotype of gliomas [37]. Furthermore, inhibition of TGF-*β* signaling restores immune surveillance and is associated with improved survival in a glioma model [37].

We previously reported the immunotherapeutic properties of interleukin-2 secreting syngeneic/allogeneic cells in the treatment of brain tumors in mice [38]. Mice with an intracerebral (i.c.) glioma treated solely by intratumor injections with allogeneic cells genetically modified to secrete IL-2 survived significantly longer than mice in various control groups. The antitumor response was mediated predominantly by CD8^+^ T cells and NK/LAK cells [39]. Intratumoral injections of the cytokine-secreting cells resulted in the killing of only the neoplastic cells; nonneoplastic cells were unaffected. Of special interest, mice injected intracerebrally with the cytokine-secreting allogeneic cells alone exhibited no neurologic deficit and there was no adverse effects on survival. The injection of IL-2 secreting allogeneic cells into the microenvironment of an i.c. tumor induced an antitumor immune response capable of prolonging survival.

In another study, the possible benefits of combining administration of the chemotherapeutic agent paclitaxel with immunotherapy in the treatment of C3H/He mice bearing an established highly aggressive intracerebral breast cancer was explored [40]. Paclitaxel is a widely-used chemotherapeutic agent which is known to induce apoptosis, although the mechanism of action is poorly understood [41]. The mice were treated by injection into the tumor bed with the DNA-based vaccine, with paclitaxel administered intraperitoneally or by paclitaxel followed by immunization with the DNA-based vaccine. The results indicated that the survival of mice with an established intracerebral breast cancer was prolonged by treatment with either paclitaxel or the DNA-transfected fibroblasts (*P* < .025), but survival of mice receiving the combined therapy did not exceed that of tumor-bearing mice receiving either form of treatment alone. The suppression of the peripheral white blood cell count attributed to paclitaxel, although relatively brief, makes paclitaxel along with most chemotherapeutic agents somewhat antagonistic when administered with immunotherapeutic treatment strategies. Nevertheless, the combination of systemic chemotherapy along with immunotherapy has been used to treat patients with advanced-stage carcinoma [42]. It has been proposed that dying tumor cells, particularly those killed by chemotherapy, engage with antitumor immune responses [43].

Since the tumor cell population is known to be heterogeneous and includes cells that are resistant to cellular immune mechanisms, it is likely that there is a subpopulation of tumor cells that are resistant to host immune mechanisms. In order to control tumor growth, a combination of therapeutic strategies will be required. Although thiazolidinediones reduce the antigen presenting capacity of dendritic cells along with reducing T cell proliferation and cytokine secretion, TZDs do not suppress the immune system and bone marrow in the same fashion as typical chemotherapeutic agents such that these agents should not compete with immune therapeutic strategies in the treatment of various tumors. In addition, their metabolic effects would be expected to occur independent of cell origin.

To test this, we carried out studies in C57Bl/6 mice with an established glioma [44]. The results of that study showed that oral pioglitazone was not effective in prolonging survival in mice bearing a highly malignant glioma (Gl261) grown intracerebrally in syngeneic C57Bl/6 mice. Intracerebral injection of pioglitazone was effective in prolonging survival in mice with an intracerebral glioma. Furthermore, intracerebral injection of fibroblasts genetically engineered to secrete IL-2 into an established intracerebral glioma was effective both in prolonging survival and stimulating a systemic antitumor immune response as measured in the spleen cells using an IFN-*γ* ELISPOT assay. In previous studies, it was confirmed that the antitumor immune responses in the tumor bearing mice were mediated predominantly by CD8^+^ and NK/LAK cells [39]. However, there was a synergistic response in prolonging survival of the animals with an established intracerebral glioma treated with both IL-2 secreting fibroblasts and pioglitazone. It should be noted nevertheless that splenic T cells isolated from mice treated with both IL-2 secreting cells and pioglitazone showed no increase in response as compared with the spleen cells isolated from animals treated with IL-2 secreting fibroblasts alone as measured by the ELISPOT IFN-*γ* assay. These results suggest that the tendency of pioglitatzone to increase the mean survival time in mice bearing a glioma is not due to an increase in systemically driven T cell immunity against the Gl261 cells.

The above results suggest that treatment with TZDs can syngergize with immunotherapeutic approaches to increase glioma cell death. While the mechanisms involved remain to be elucidated, it is likely to be a combination of events including a reduction in the immunosuppressive responses of the tumor cells leading to an increase in cytotoxic T cell numbers or activity as well as a decrease in tumor cell mitochondrial function making the cells more susceptible to induction of apoptosis by cytokines.
